# Excessive phospholipid peroxidation distinguishes ferroptosis from other cell death modes including pyroptosis

**DOI:** 10.1038/s41419-020-03118-0

**Published:** 2020-10-27

**Authors:** Bartosz Wiernicki, Hanne Dubois, Yulia Y. Tyurina, Behrouz Hassannia, Hülya Bayir, Valerian E. Kagan, Peter Vandenabeele, Andy Wullaert, Tom Vanden Berghe

**Affiliations:** 1 VIB Center for Inflammation Research, Ghent, Belgium; 2 Department of Biomedical Molecular Biology, Ghent University, Ghent, Belgium; 3 Department of Internal Medicine and Pediatrics, Ghent University, Ghent, Belgium; 4 Center for Free Radical and Antioxidant Health, Department of Environmental Health and Occupational Health, University of Pittsburgh, Pittsburgh, PA 15213 USA; 5 Department of Biomedical Sciences, University of Antwerp, Antwerp, Belgium; 6 Children’s Neuroscience Institute, UPMC Children’s Hospital of Pittsburgh, Pittsburgh, PA 15224 USA; 7 Safar Center for Resuscitation Research, Department of Critical Care Medicine, University of Pittsburgh, Pittsburgh, PA 15224 USA; 8 Institute for Regenerative Medicine, Sechenov First Moscow State Medical University, 119991 Moscow, Russia; 9 Methusalem program; Ghent University, Ghent, Belgium

**Keywords:** Lipidomics, Cell death

## Abstract

Lipid peroxidation (LPO) drives ferroptosis execution. However, LPO has been shown to contribute also to other modes of regulated cell death (RCD). To clarify the role of LPO in different modes of RCD, we studied in a comprehensive approach the differential involvement of reactive oxygen species (ROS), phospholipid peroxidation products, and lipid ROS flux in the major prototype modes of RCD viz. apoptosis, necroptosis, ferroptosis, and pyroptosis. LC-MS oxidative lipidomics revealed robust peroxidation of three classes of phospholipids during ferroptosis with quantitative predominance of phosphatidylethanolamine species. Incomparably lower amounts of phospholipid peroxidation products were found in any of the other modes of RCD. Nonetheless, a strong increase in lipid ROS levels was detected in non-canonical pyroptosis, but only during cell membrane rupture. In contrast to ferroptosis, lipid ROS apparently was not involved in non-canonical pyroptosis execution nor in the release of IL-1β and IL-18, while clear dependency on CASP11 and GSDMD was observed. Our data demonstrate that ferroptosis is the only mode of RCD that depends on excessive phospholipid peroxidation for its cytotoxicity. In addition, our results also highlight the importance of performing kinetics and using different methods to monitor the occurrence of LPO. This should open the discussion on the implication of particular LPO events in relation to different modes of RCD.

## Introduction

Up to this day, multiple types of regulated cell death (RCD) have been identified^1^. Excessive lipid peroxidation (LPO) of polyunsaturated fatty acid (PUFA) containing cell membrane phospholipids (PLs) has originally been conceptualized as a hallmark of ferroptosis—an iron-catalyzed mode of necrotic cell death^2^. Most ferroptosis inducers rely on blocking the activity of glutathione peroxidase 4 (GPX4)—an enzyme able to reduce PL-hydroperoxide (PL-OOH) to its alcohol form (PL-OH). Consequently, PL-OOH upon reaction with labile iron generates alkoxyl and peroxyl radicals, which further fuels oxidation of PLs (oxPLs) in cellular membranes^3^. The continued oxidation of PUFA-containing PLs is followed by thinning and increased curvature of membrane, thereby stimulating oxidative micellization, pore formation, and subsequent cell membrane rupture^4^.

Aside from ferroptosis, some specific LPO events have also been described in apoptosis^5^, necroptosis^6^, and pyroptosis^7,8^. Those studies, however, differ in terms of used models and detection methods for LPO. To clarify the role of LPO, in particular oxPLs, in different RCDs, we subjected bone-marrow-derived macrophages (BMDMs) to apoptosis, necroptosis, ferroptosis as well as canonical and non-canonical pyroptosis and performed liquid chromatography-mass spectrometry-based redox-lipidomics analysis, further coined as the oxidative lipidomics approach, a gold standard method to identify particular oxidized lipid species^9^. Additionally, we utilized the properties of BODIPY™ 581/591 (C11-BODIPY) and dihydrorhodamine 123 (DHR123) probes that change their fluorescence properties upon oxidation. Oxidation of the polyunsaturated butadienyl portion of C11-BODIPY results in a shift of the fluorescence emission peak from ~590 nm to ~510 nm. C11-BODIPY resides in lipophilic membrane structures where it can be oxidized by reactive enzymatic and/or non-enzymatic pro-oxidant intermediates, including alkoxyl and peroxyl radicals. Its oxidation is therefore an indirect manifestation of the lipid ROS flux^10^, further coined as lipid ROS. DHR123 on the other hand has been used to measure intracellular reactive oxygen species (ROS) levels particularly hydrogen peroxide (in the presence of peroxidases) and oxidants derived from peroxynitrite^11^. To the best of our knowledge, this is the first systematic study that compares accumulation of oxidized phospholipids (oxPLs), ROS and lipid ROS by various methods on different modes of RCD.

## Results

### Predominant PL peroxidation products are detected in ferroptosis, while incomparably lower levels in apoptosis, necroptosis, and pyroptosis

**Fig. 1:**
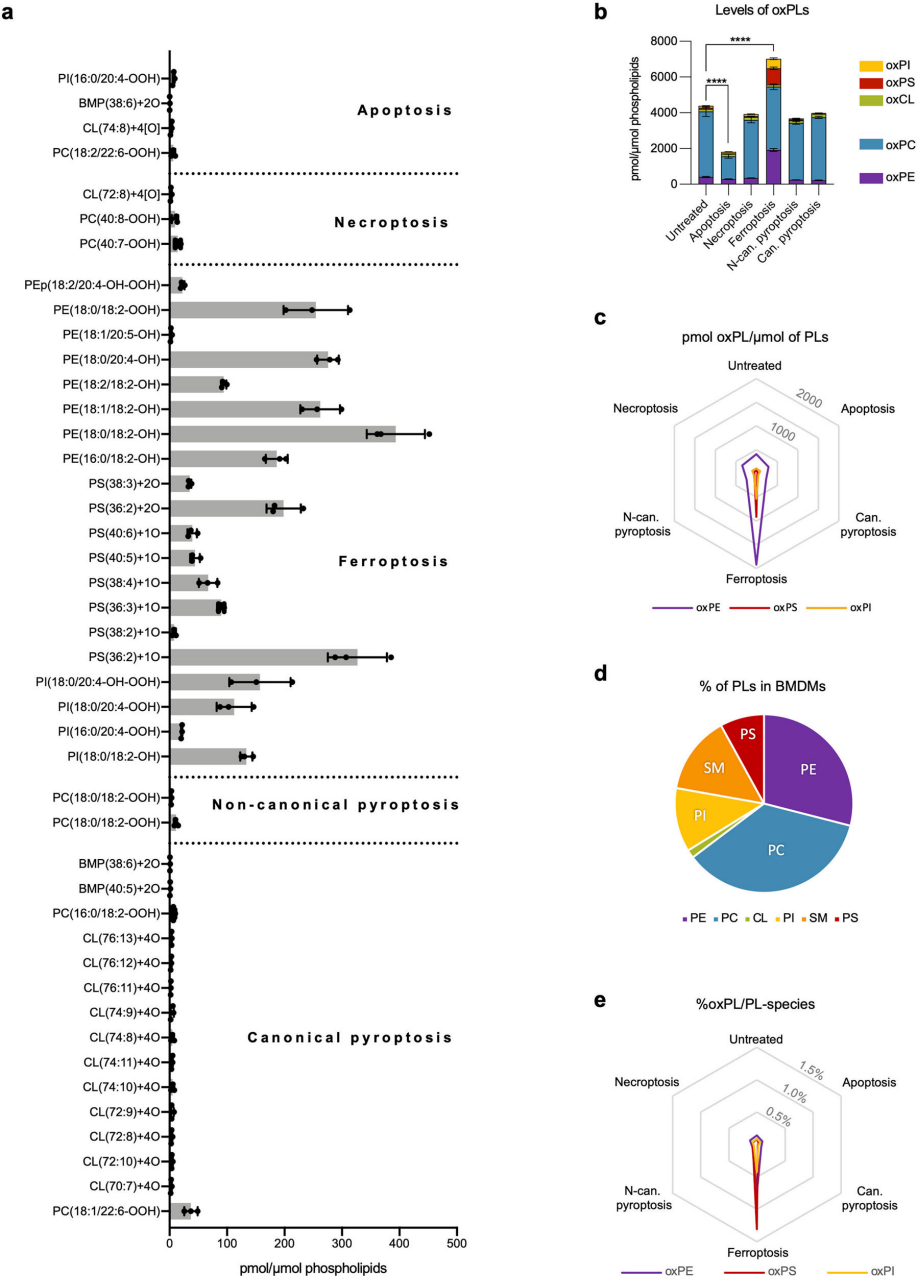
Oxidative lipidomics analysis of BMDMs during apoptosis, necroptosis, ferroptosis and pyroptosis. **a** “Oxidative lipidomics” of BMDMs subjected to apoptosis (4 h, 4000IU/ml hTNF and 5 μM TPCA-1), necroptosis (6 h, 4000IU/ml hTNF, 5 μM TPCA-1 and 10 μM zVAD.fmk), ferroptosis (4 h, 0.5 μM ML162), canonical pyroptosis (3 h of 1 µg/ml LPS followed by 30 min incubation with 5 mM ATP) and non-canonical pyroptosis (3 h of 1 µg/ml LPS followed by 4 h incubation after LPS transfection). Only oxidized lipid species statistically different from untreated samples are plotted. **b** Level of oxPLs analyzed by ‘oxidative lipidomics’ in BMDMs subjected to different types of RCD. **c** Level of oxPE, oxPS and oxPI in different modes of cell death. **d** Percentage of different groups of PL in untreated BMDMs. **e** Percentage of oxPLs relative to their unoxidized counterparts. The combined results of three biological replicates are shown. **a, b** Bars are mean ± SEM, one-way ANOVA, **p < 0.01, ****p < 0.0001.

To enable a comprehensive side by side comparison of the role of oxPLs in the different modes of RCD in a single cell type, we chose to work with primary BMDMs because, depending on the trigger, these cells are able to undergo apoptosis, necroptosis, pyroptosis and ferroptosis (Supplementary Fig. 1a). To determine the role of oxPLs in the execution of different modes of RCD, we subjected BMDMs at the early stage of cell death (Supplementary Fig. 1b) to oxidative lipidomics to evaluate in an unbiased way oxPLs events preceding cell membrane permeabilization. While specific oxPLs were detected in all types of RCD compared to untreated samples, a magnitude higher level of oxPLs was observed in ferroptotic cells (Fig. 1a, b). Qualitative differences between samples confirmed the unique profile of excessive oxPLs in ferroptosis. While in apoptotic, necroptotic, and pyroptotic BMDMs mostly oxidation of cardiolipin and/or phosphatidylcholine were detectable (Fig. 1a), BMDMs undergoing ferroptosis revealed mainly oxidized phosphatidylethanolamine (oxPE) species followed by oxidized phosphatidylserine (oxPS) and phosphatidylinositol (oxPI) (Fig. 1c). The levels of oxidized phosphatidylcholine and cardiolipin in ferroptosis-stimulated cells were not significantly different from untreated samples (Fig. 1a). Remark that the overrepresentation of oxPE could be due to its high relative abundance compared to PS and PI (Fig. 1d). When the relative oxidation rate per PL-species is calculated, PS is relatively highest oxidized with oxPS representing up to 1.30% (Fig. 1e). Although, the comprehensive oxidative lipidomics analysis in BMDMs revealed specific oxPLs events in all prototypes of RCD, it is obvious that the extend PL oxidation is a scale higher during ferroptosis as reflected by the oxidation levels of mainly three PL-species (PS, PI, and PE). Therefore, we propose to use the term “excessive PL peroxidation” to refer to this extensive signature of oxPLs during ferroptosis.

### Increase in lipid ROS is an early event during ferroptosis, while a late event during cell membrane rupture in non-canonical pyroptosis

**Fig. 2:**
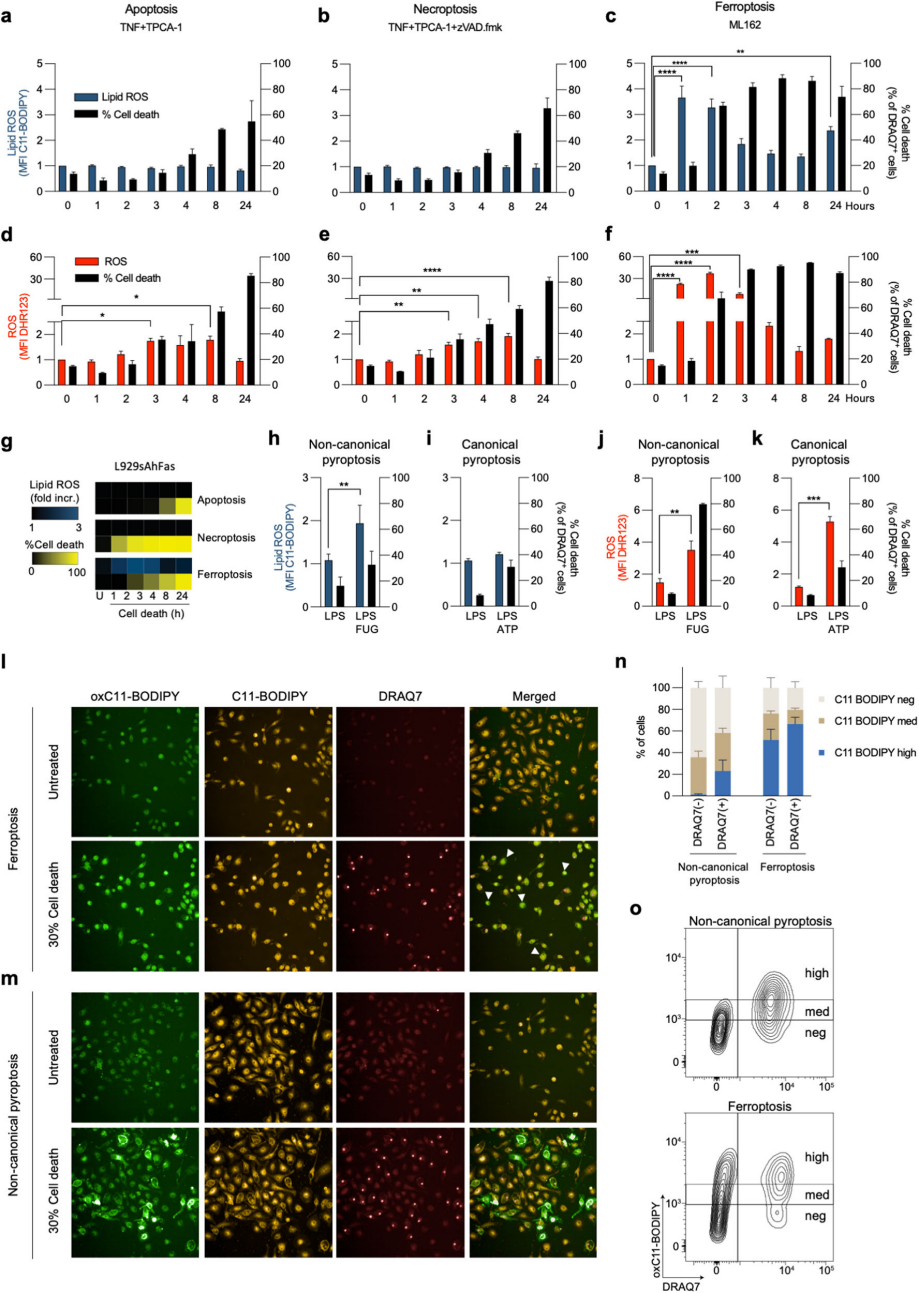
Analysis of ROS and lipid ROS during apoptosis, necroptosis, ferroptosis, and pyroptosis. **a**–**c** Lipid ROS production during apoptosis (4000IU/ml hTNF and 5 μM TPCA-1), necroptosis (4000IU/ml hTNF, 5 μM TPCA-1 and 10 μM zVAD.fmk) and ferroptosis (0.5 μM ML162) in BMDMs. **d**–**f** ROS levels during apoptosis, necroptosis, and ferroptosis in BMDMs. **g** Lipid ROS and cell death in L929sAhFas cells in different modes of RDC, heatmaps represent mean values from three independent experiments. **h**–**i** Lipid ROS during non-canonical (3 h of 1 µg/ml LPS followed by 8 h incubation after LPS transfection) and canonical (3 h of 1 µg/ml LPS followed by 30 min incubation with 5 mM ATP) pyroptosis. **j**–**k** ROS during canonical and non-canonical pyroptosis. **l**–**m** Representative fluorescent microscopy images of BMDMs undergoing ferroptosis and non-canonical pyroptosis reaching ~30% of cell death, oxC11-BODIPY (oxidized) represents the levels of lipid ROS, while C11-BODIPY represents the level of staining with the probe (unoxidized), DRAQ7 was used as a cell membrane permeabilization marker. **n** Analysis of C11-BODIPY signal intensity during ferroptosis and non-canonical pyroptosis based on flow cytometry experiments. **o** Representative contour plots during flow cytometry measurements of ferroptotic (3 h) and pyroptotic (8 h) BMDMs with stratification of the C11-BODIPY signal. All bars are mean ± SEM of at least three biological replicates. One-way ANOVA, *, *p* < 0.05, **p < 0.01, ***p < 0.001, ****p < 0.0001.

C11-BODIPY is generally used to monitor the flux of lipid ROS in cell membranes, which is assumed to be associated with a pro-oxidant activity and excessive PL peroxidation e.g. during ferroptosis. In order to complement the above oxidative lipidomics fingerprints, we performed a time-course analysis to monitor both ROS and lipid ROS in all prototype modes of RCD in BMDMs. Using a standardized gating strategy on non-permeabilized cells (Supplementary Fig. 2a), we did not detect an increase in lipid ROS in apoptosis and necroptosis (Fig. 2a, b), while we observed a ~2-fold increase in ROS (Fig. 2d, e). In sharp contrast, during ferroptosis both an increase in ROS (up to 35-fold) and lipid ROS (4-fold) was observed, which preceded cell death (Fig. 2c, f). Similar results were obtained for fibroblast L929sAhFas cells subjected to apoptosis, necroptosis, and ferroptosis (Fig. 2g, Supplementary Fig. 2b–g). Unexpectedly, we detected increased levels of lipid ROS in non-canonical, but not in canonical pyroptosis (Fig. 2h, i) despite increase of ROS (4-/5-fold) in both of them (Fig. 2j, k). Live cell imaging revealed a differential lipid ROS signature viz. during ferroptosis an early homogeneous staining pattern preceding cell membrane rupture (Fig. 2l and Supplementary Fig. 2h), and during non-canonical pyroptosis a late patchy staining pattern is observed coinciding with the cell membrane rupture (Fig. 2m and Supplementary Fig. 2i). Similarly, flow cytometric analysis revealed high levels of lipid ROS prior to cell membrane permeabilization during ferroptotic, but not pyroptotic cells (Fig. 2n, o). Overall, these data indicate an increased signature of pro-oxidant activity (oxidative stress) in all prototype forms of RCD, while a strong increase in lipid ROS is only detected at the early phase of ferroptosis induction. During non-canonical pyroptosis however, we only detected an increase in lipid ROS signal at the late phase coinciding with cell membrane rupture. This suggests that specific peroxy-PL pro-death signals are only generated in ferroptotic cells.

### Lipid ROS during non-canonical pyroptosis is dependent on CASP11 and GSDMD

**Fig. 3:**
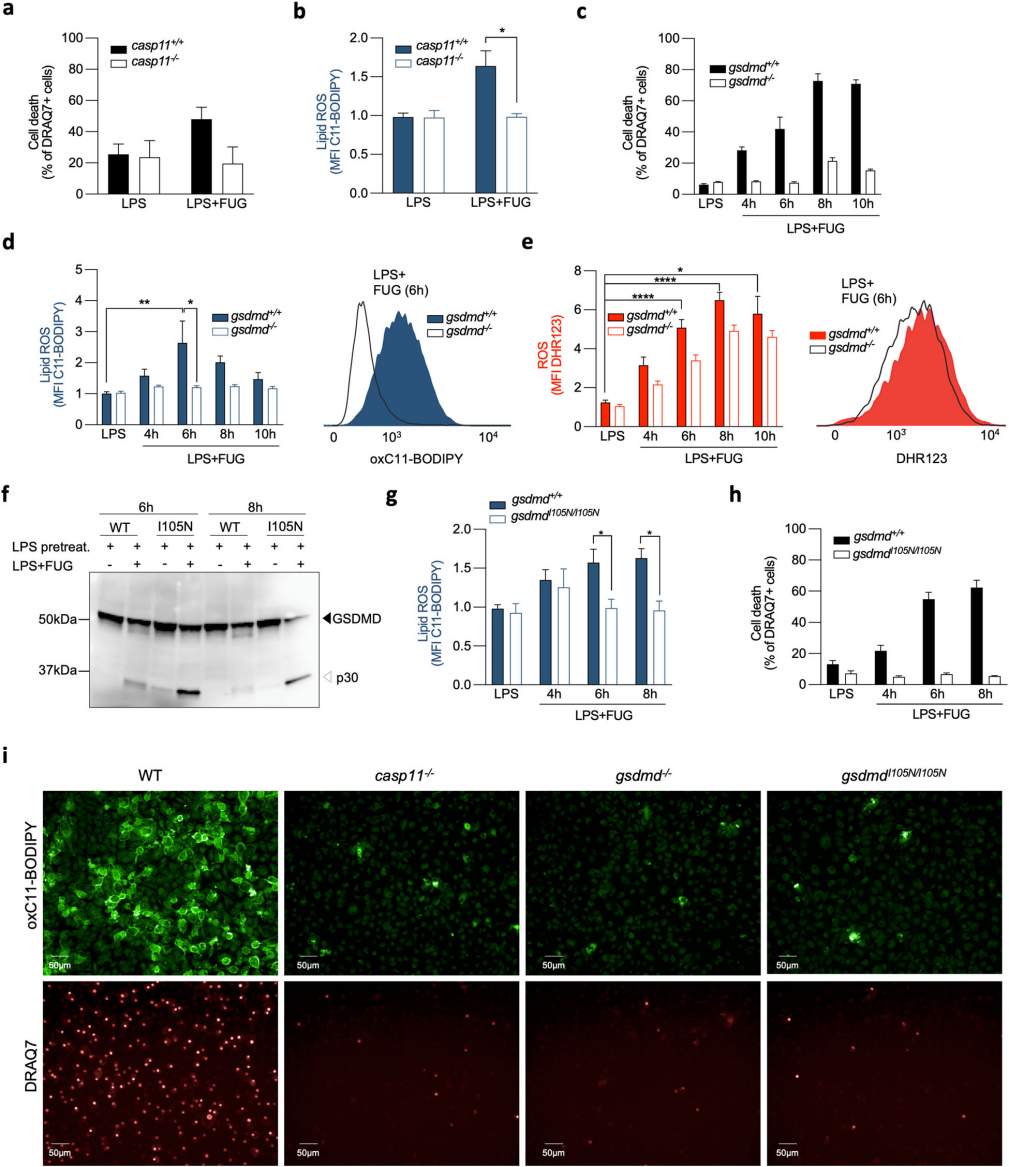
Analysis of ROS and lipid ROS during non-canonical pyroptosis in genetic models targeting the pyroptosis pathway. **a**–**b** Cell death and lipid ROS accumulation in *casp11*^−/−^ BMDMs and WT 8 h after LPS transfection of LPS-primed cells. **c**–**e** Cell death, lipid ROS and ROS in *gsdmd*^−/−^ BMDMs during non-canonical pyroptosis. **f** Cleavage of WT and *gsdmd*^I105N/I105N^. **g**–**h** Lipid ROS and cell death of *gsdmd*^I105N/I105N^ during non-canonical pyroptosis. **i** Representative fluorescent microscopy images of WT, *casp11*^−/−^, *gsdmd*^−/−^ and *gsdmd*^I105N/I105N^ stained with C11-BODIPY and DRAQ7 5 h after LPS transfection. All bars are mean ± SEM of three biological replicates. One-way ANOVA, *p < 0.05, **p < 0.01.

To clarify the possible role of lipid ROS in cell membrane rupture during non-canonical pyroptosis^7^, we utilized several genetic models of BMDMs targeting pyroptosis pathway. LPS-induced non-canonical pyroptosis is mediated by caspase-11 activation, which then cleaves gasdermin D (GSDMD) to generate the N-terminal GSDMD p30 fragment that forms cytotoxic pores in the plasma membrane^12^. *Casp11*-deficient BMDMs subjected to LPS transfection were protected from cell death (Fig. 3a) and did not show an increase in lipid ROS (Fig. 3b). Similarly, the C11-BODIPY signal remained low in *gsdmd*-deficient BMDMs (Fig. 3c, d), while ROS was hardly affected (Fig. 3e). Lipid ROS was also absent in BMDMs expressing a mutated form of GSDMD replacing *Ile* to *Asn* at position 105 (*gsdmd*^I105N/I105N^)^12^ (Fig. 3g) that undergoes cleavage (Fig. 3f) but is defective in pore formation and cell death induction (Fig. 3h). This suggests that the ability of GSDMD to create pores in the plasma membrane crucially contributes to lipid ROS during non-canonical pyroptosis. Live cell imaging further confirmed the necessity of functional CASP11 and GSDMD in generating lipid ROS during non-canonical pyroptosis (Fig. 3i).

### Lipid ROS is functionally involved in ferroptosis, but not in pyroptosis execution

**Fig. 4:**
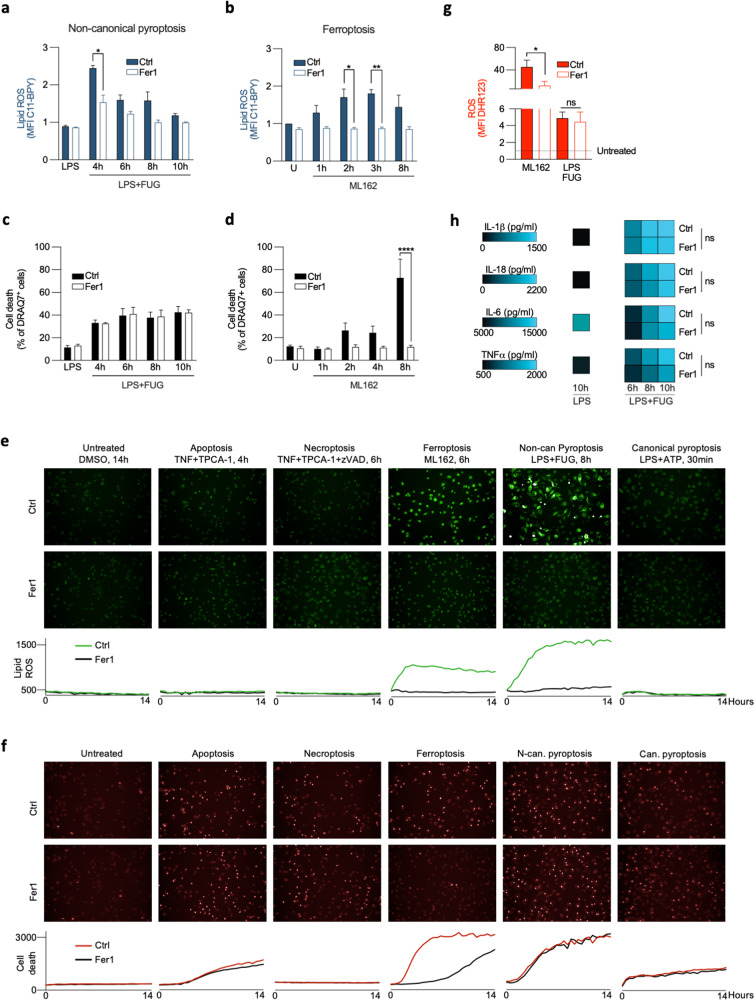
The effect of Fer1 on ferroptosis and non-canonical pyroptosis execution. **a**, **b** Lipid ROS accumulation during ferroptosis (0.5 μM ML162) and non-canonical pyroptosis (3 h of 1 µg/ml LPS followed by 8 h incubation after LPS transfection) in the presence of 0.5 µM Fer1. **c**, **d** Cell death levels during ferroptosis and non-canonical pyroptosis in the presence of Fer1. **e**–**f** Representative fluorescent microscopy images from BMDMs undergoing different modes RCD showing the impact of Fer1 on lipid ROS and cell death. **g** The impact of Fer1 on ROS production during ferroptosis and non-canonical pyroptosis. **h** The impact of Fer1 on production of pro-inflammatory cytokines. Heatmaps represent values from three biological replicates. All bars are mean ± SEM of three biological replicates. One-way ANOVA, *p < 0.05, **p < 0.01, *p* < 0.0001.

To assess the functional implication of lipid ROS or oxPLs in cell death execution, we next performed a genetic approach by overexpression GPX4 and as a pharmacological approach using the lipophilic radical trap Ferrostatin-1 (Fer1)^3^ to block LPO. While GPX4 overexpression clearly inhibited the increase in lipid ROS in ferroptotic BMDMs (Supplementary Fig. 3a, b), it failed to reduce ROS and lipid ROS during non-canonical pyroptosis (Supplementary Fig. 3c–e), suggesting that lipid ROS in this type of RCD does not originate from oxPLs. When we supplemented BMDMs undergoing ferroptosis and non-canonical pyroptosis with Fer1, we observed a decrease in lipid ROS in both cell death modes (Fig. 4a, b, e) due to scavenging capacity of Fer1 as well as competition with fluorescent probes in the oxidation process. Importantly, only BMDMs stimulated with a ferroptosis inducer were rescued from cell death (Fig. 4c, d, f). Remark that ROS was not reduced by Fer1 during non-canonical pyroptosis in contrast to ferroptosis (Fig. 4g), which suggests that ROS and lipid ROS might be mutually interlinked during ferroptosis but not during non-canonical pyroptosis. Moreover, administration of Fer1 did not impact release of pro-inflammatory cytokines from pyroptotic cells (Fig. 4h). In line with the observation that lipid ROS is not increased in apoptosis, necroptosis, and canonical pyroptosis, Fer1 did not have any impact on the cell death execution (Fig. 4e, f). In conclusion, an early increase in lipid ROS is involved in execution of ferroptosis resulting in excessive generation of oxPLs, but not in apoptosis, necroptosis, and pyroptosis.

## Discussion

To the best of our knowledge, a comparative study on the presence and role of excessive LPO in different modes of RCD has not been reported. While LPO is the major executioner mechanism in ferroptosis, it has also been linked to apoptosis^5^, necroptosis^6^ and more recently to pyroptosis^7,8^. Utilizing an unbiased oxidative lipidomics approach, we showed (per)oxidation of PLs in all modes of RCD with by far the highest levels of oxPL (mainly oxPS, oxPE and oxPI) in ferroptosis. The commonly used probe C11-BODIPY showed high increase in lipid ROS during ferroptosis, but unexpectedly also during non-canonical pyroptosis. While Fer1 blocks lipid ROS during both ferroptosis and non-canonical pyroptosis, in contrast to ferroptosis it does not affect non-canonical pyroptosis suggesting that increase in lipid ROS is not implicated in the pyroptosis execution.

It has been proposed that oxidation of PLs in ferroptosis occurs in semi-specific manner making some oxPL more prone to the oxidative process than others. PE has been identified as a crucial PL oxidized during ferroptosis^13^. However other PLs are also oxidized during ferroptosis^13,14^. In line with these findings, we detected oxPE, oxPS and oxPI during ferroptosis in BMDMs. The high levels of oxPS (1.30%) and lack of any oxPC despite its dominant presence in BMDMs suggests also here some specificity in the process. The length, number, and position of double bounds in the lipid tails of the PLs are likely determining factors in the progression and specificity of PL oxidation.

Although lipid ROS typically initiates LPO, we detected low only levels of two oxPLs in non-canonical pyroptosis, at least at early stage of cell death induction (25%). Our results show that C11-BODIPY fluorescence increase coincides with cell membrane permeabilization and seems to occur downstream of GSDMD-N pore formation. The necessity of cleaved GSDMD in generating lipid ROS may lay in its ability to permeabilise not only plasma membrane, but also intracellular organelles including mitochondria^15^. Note also that live cell imaging showed a granular intracellular C11-BODIPY staining during non-canonical pyroptosis, in contrast to a homogenous staining pattern during ferroptosis. This might hint towards a different source or mode of action of lipid ROS. Alternatively, oxidation of C11-BODIPY probe might not fully reflect the state of membrane PL oxidation, as it was demonstrated that C11-BODIPY may be more prone to oxidation compared to lipids physiologically present in cellular membranes^16^.

The differential LPO induction pending on the type of pyroptosis may also have differential effects on the innate immune system. Indeed, oxidized lipids have been associated with anti-inflammatory signaling in macrophages^17^. Moreover, caspase-11 dependent, but not caspase-1 dependent pyroptosis has been shown to induce ferritin release from dying macrophages^18^. Ferritin serves as an iron storage protein protecting cells from iron-catalyzed oxidative stress by buffering the labile iron pool (LIP)^19^. This drop of ferritin in dying BMDMs during non-canonical pyroptosis might result in an increase LIP boosting lipid ROS generation during the final stages of cell rupture.

In line with our in vitro observations, Fer1 also failed to rescue the observed sensitization of macrophage-specific *gpx4*-deficient mice in model of cecal ligation and puncture-induced shock^8^. One could expect that the absence of GPX4 in macrophages sensitizes them for lipid ROS and cell death at site of infection resulting in worsening of the outcome. Recently, a role for ALOX5-mediated LPO was proposed in non-canonical pyroptosis upstream of inflammasome activation^7^. This might imply that enzymatic, but not non-enzymatic LPO is involved in non-canonical pyroptosis signaling. Note in this respect that C11-BODIPY is generally used to detect non-enzymatic, excessive lipid ROS in cellular membranes rather than particular enzymatic LPO. Additionally, Fer1 has been shown to have limited ability to inhibit enzymatically driven LPO^20^. While excessive lipid ROS does not have a role in non-canonical pyroptosis induction, it cannot be excluded that specific, enzymatic LPO may occur at the early stages of cell death and, similarly to apoptosis^5^, be indispensable for cell death progression.

C11-BODIPY has been described as a reliable tool to measure excessive LPO in cells using flow cytometry and fluorescent microscopy^21^, even though it does not reach the specificity and sophistication of oxidative lipidomics^22^. So far, C11-BODIPY has been used mostly in the context of ferroptosis and can be useful in differentiating ferroptosis from necroptosis and apoptosis. However, we advise some caution using it in a more complex context of inflammation in in vivo and in vitro because of the positivity of C11-BODIPY in non-canonical pyroptotic death cells, which can be circumvented by gating on living cells. In addition, functional studies can be an extra strategy to clarify the involvement of excessive LPO in the dying process by analyzing the ability of lipophilic antioxidants to block lipid ROS, and subsequent death. Finally, our data also show that excessive generation of lipid ROS and oxPLs, which is inhibitable by Fer1, is a hallmark of ferroptosis differentiating it from apoptosis, necroptosis, and pyroptosis.

## Materials and methods

### Mice

Casp11^−/−^ mice were generated by sequentially breeding parental Casp4^tm1b(KOMP)Wtsi^ mice (KOMP ID CSD47499) with Flp-deleter mice to remove the Frt site flanked LacZ reporter and Neomycin selection cassette, and with Cre-deleter mice to remove LoxP site flanked exons 3 and 4. Gsdmd^−/−^ mice and Gsdmd^I105N^ mutant (C57BL/6Janu-Gsdmdm1Anu/AnuApb) mice were kindly provided by Dr. Vishva M. Dixit (Genentech) and the Australian Phenomics Facility at the Australian National University, respectively^12^. All mice were bred in specific pathogen-free animal facilities and were females between 8 and 12 weeks during all the experiments. Mice were housed in air-conditioned, temperature-controlled rooms with 14/10–h light/dark cycles. Food and water were provided ad libitum. C57BL/6J WT mice were purchased from Janvier (Le Genest, France). All mice experiments were organized in accordance with institutional, national, and European animal regulations.

### Cell culture conditions

Primary macrophages were generated from bone-marrow cells flashed out from femur and tibia. Cells were differentiated to macrophages by culturing them in Iscove’s modified Dulbecco’s medium (IMDM, Lonza) with 10% (vol/vol) FBS, 30% (v/v) L929 cell-conditioned medium, penicillin (100U/ml) and streptomycin (100 mg/ml) for 6 days in 37 °C in the presence of 5% CO_2_. After the differentiation, cells were detached using LPS free 10 mM EDTA/PBS solution for specific experiments. L929sAhFas cells were cultured in DMEM medium supplemented with 10% of FBS (v/v) and l-glutamine (0.03%). Cells were cultured in 37 °C in a humidified atmosphere containing 5% CO_2_ and split every 3–4 days using trypsin/EDTA solution.

### Cell death analysis and cytokine measurements

Cell death was analyzed according to previously published protocol^23^. Briefly, cells were seeded in 96-well plate (10000/well) and stimulated for cell death next day. For L929sAhFas cells, apoptosis was induced by administration of 125 ng/ml human anti-Fas antibody (clone CH-11, BioConnect B.V., Huissen, Netherlands); necroptosis was induced by 5000IU/ml of mouse TNF (VIB Protein Service Facility, Gent, Belgium) and—optionally—10 μM zVAD.fmk (VWR International, PA, USA); ferroptosis was induced by 10 μM of ML162 (Aobious, Gloucester, MA, USA). For BMDMs, apoptosis was induced by administration of 4000IU/ml hTNF (VIB Protein Service Facility) and 5 μM TPCA-1 (Tocris Bioscience, Bristol, UK), while necroptosis was induced by additional supplementation with 10 μM zVAD.fmk. Ferroptosis was induced by 0.5 μM ML162. After adding 2.5 μM SytoxGreen (Molecular Probes, USA) and 33 μM DEVD-amc (Peptanova, Sandhausen, Germany) fluorescence was measured every 1–2 h using FLUOstar Omega (BMG Labtech). Cell death was calculated based on fluorescence from 100% dead cells, killed by 0.5% of TritonX. Pyroptosis in primary macrophages was induced by 3 h priming cells with 1 µg/ml LPS, which was followed either by addition of 5 mM ATP (canonical pyroptosis) or LPS transfection using FuGENE^®^ reagent (Promega, Madison, WI, USA) (non-canonical pyroptosis). The mix for transfection was prepared by 15 min incubation of 2 µg/ml of LPS with FuGENE^®^ according to the manufacturer’s protocol in room temperature. Mouse cytokines in cell culture supernatants were determined by magnetic bead-based multiplex assay using Luminex technology (Bio-Rad, Hercules, CA, USA).

### Oxidative lipidomics

Lipids were extracted by Folch procedure^24^ with slight modifications, under nitrogen atmosphere, at all steps. Lipid phosphorus was determined by a micro-method^25^ LC/ESI-MS analysis of lipids was performed on a Dionex HPLC system coupled to an Orbitrap Fusion Lumos mass spectrometer (ThermoFisher Scientific). PLs were separated on a normal phase column (Luna 3 μm Silica (2) 100 A, 150 × 1.0 mm, (Phenomenex) at a flow rate of 0.050 ml/min. The column was maintained at 35 °C. The analysis was performed using gradient solvents (A and B) containing 10 mM ammonium format. Solvent A contained propanol:hexane:water (285:215:5, v/v/v) and solvent B contained propanol:hexane:water (285:215:40, v/v/v). The column was eluted for 0.5 min isocratically at 25% B, then from 0.5 to 6.5 min with a linear gradient from 25 to 40% solvent B, from 6.5 to 25 min using a linear gradient from 40 to 55% solvent B, from 25 to 38 min with a linear gradient from 55 to 70% solvent B, from 38 to 48 min using a linear gradient from 70 to 100% solvent B, then isocratically from 48 to 55 min at 100% solvent B followed by a return to initial conditions from 55 to 70 min from 100 to 25% B. The column was then equilibrated at 25% solvent B for an additional 5 min. The mass spectrometer was operated with electrospray ionization probe in negative polarity mode. Ion source conditions were set as follows: Spray voltage = 3 kV, Sheath gas = 55 (arbitrary unit), Auxiliary gas = 10 (arb. unit), Sweep gas = 0.5 (arb. unit), Transfer tube temperature = 300 °C, Vaporizer temperature = 200 °C, RF-Lens level = 20%. Analysis of LC/MS data was performed using software package Compound Discoverer™ (ThermoFisher) with an in-house generated analysis workflow and oxidized PL database. Lipids were further filtered by retention time and confirmed by fragmentation mass spectrum. Deuterated PLs (Avanti Polar Lipids) were used as internal standards.

### Flow cytometry measurements

L929sAhFas cells and BMDMs were seeded in 24-well suspension plates (0.1mln/ml and 0.4mln/ml accordingly) and stimulated for cell death. One hour before each time point, fluorescent probes were added to proper wells: 0.5 μM C11-BODIPY (ThermoFisher, MA, USA) or 1 μM DHR-123 (Cayman Chemical, MI, USA) and 0.5 μM of DRAQ7 cell death stain (BioStatus, Shepshed, UK). Lipid ROS and ROS accumulation was measured at specific time points using BDFortessa. Fluorescence was collected in B530 (C11-BODIPY and DHR123) and R780 (DRAQ7) channels. Only fluorescence of not permeabilized cells was analyzed. Gating strategy is presented in supplementary materials (Supplementary Fig 1).

### Microscopy experiments

BMDMs were seeded in 96-well plates (CellCarrier-96 ultra microplates, PerkinElmer, Waltham, MA, USA). Next day, C11-BODIPY (2 µM) and DRAQ7 (0.5 µM) were added to each well 15 min before measurements. After adding cell death inducers and Fer1, cell death was measured for 14 h every 30 min using OPERA microscope using 20x magnification. Data were analyzed based on mean fluorescence intensity of C11-BODIPY and DRAQ7 dyes.

### Western blot analysis

Cells were denatured in Laemmli buffer by boiling for 10 min. Separation of proteins was performed by SDS-PAGE and proteins were transferred to nitrocellulose membrane (Thermo Scientific) with semi-dry blotting. Membrane was blocked using 5% non-fat dry milk solution in TBS buffer with 0.05% Tween20 (TBST). Incubation with primary antibody against GPX4 (1:1000; Abcam ab125066), actin (1:5000, Bio-Connect B.V., Huissen, Netherlands) and GSDMD antibody recognizing the cleaved form^26^ was performed O/N at 4 °C in TBST. After extensive washing, the membranes were incubated with HRP-conjugated secondary anti-rabbit (1:5000; VWR International, Oud-Hevererle, Belgium, NA934), anti-mouse (1:5000; VWR International, NA931) for 1 h in RT. Membranes were developed using Western Lighting Enhanced Chemiluminescence Substrate (Perkin Elmer).

### Statistical analysis

For the analysis of oxPLs during cell death, ROS and lipid ROS measurement as well as cytokine measurements, minimum three biological replicates were used. For oxPL, lipid ROS, and ROS, data were analyzed by ordinary one-way ANOVA with Tukey test for multiple comparison. Data analysis of flow cytometry experiment was performed using the FlowJo 10.7 software.

## Supplementary information

Supplementary Figure 1
