# Supplementary figures and images for: Excessive phospholipid peroxidation distinguishes ferroptosis from other cell death modes including pyroptosis
Source: Cell Death Dis. 2020 Oct 27;11(10):922. doi: 10.1038/s41419-020-03118-0 (PMC7591475; doi:10.1038/s41419-020-03118-0)

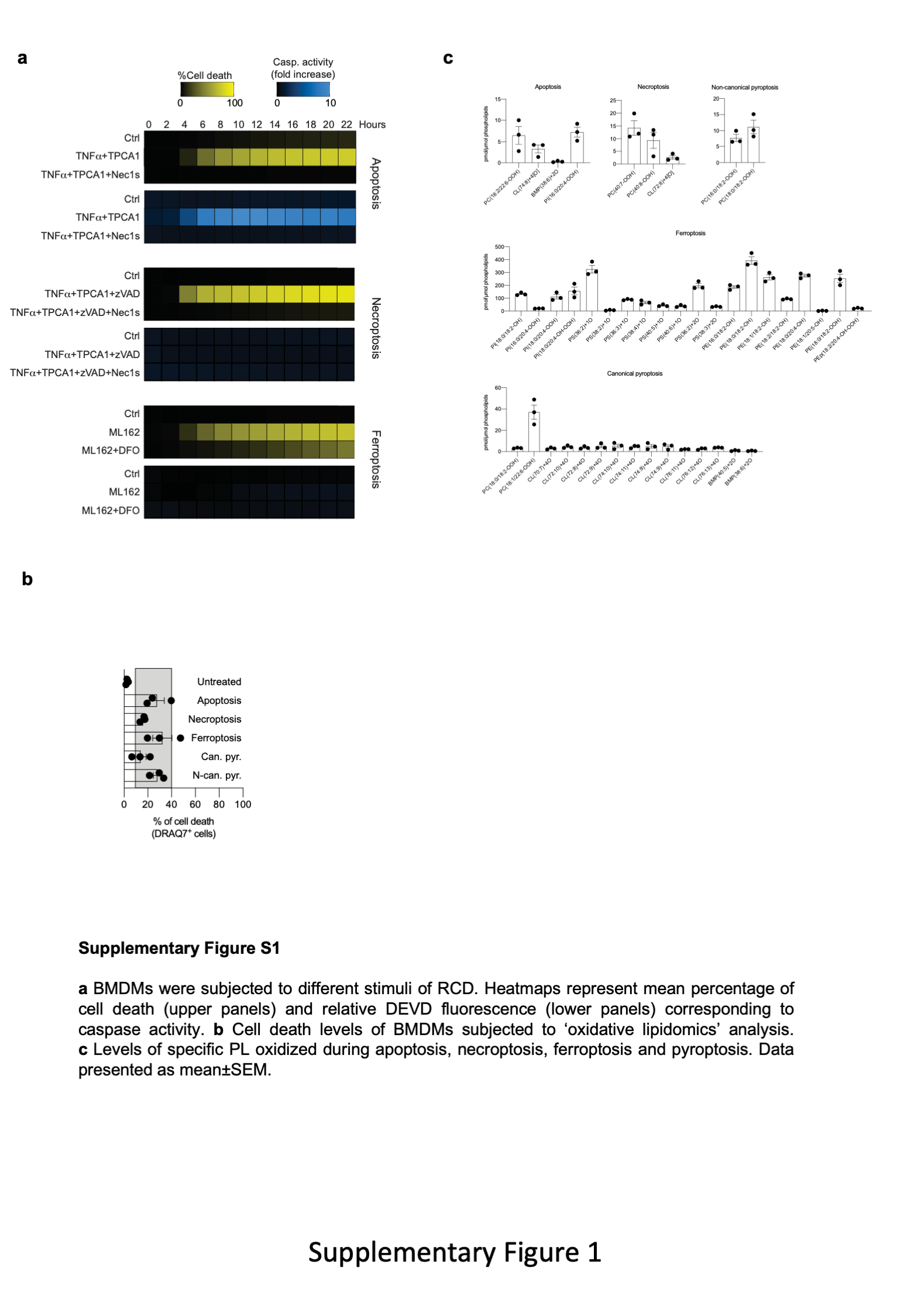

Supplement: Supplementary file 1 — Supplementary Figure 1 [file 41419_2020_3118_MOESM1_ESM.tif]
